# Identification of Molecular Targets and Potential Mechanisms of Yinchen Wuling San Against Head and Neck Squamous Cell Carcinoma by Network Pharmacology and Molecular Docking

**DOI:** 10.3389/fgene.2022.914646

**Published:** 2022-07-06

**Authors:** Biyu Zhang, Genyan Liu, Xin Wang, Xuelei Hu

**Affiliations:** ^1^ Key Laboratory of Green Chemical Engineering Process of Ministry of Education, Hubei Key Laboratory of Novel Reactor and Green Chemical Technology, School of Chemical Engineering and Pharmacy, Wuhan Institute of Technology, Wuhan, China; ^2^ School of Medicine, Jiujiang University, Jiujiang, China

**Keywords:** Yinchen Wuling San, head and neck squamous cell carcinoma, network pharmacology, target, molecular docking

## Abstract

Head and neck squamous cell carcinoma (HNSCC) represents one of the most malignant and heterogeneous tumors, and the patients have low 5-year survival. Traditional Chinese medicine (TCM) has been demonstrated as an effective complementary and/or alternative therapy for advanced malignancies including HNSCC. It has been noted that several herbs that are used for preparing Yinchen Wuling San (YWLS) have anti-tumor activities, whereas their mechanisms of action remain elusive. In this study, network pharmacology and molecular docking studies were employed to explore the underlying mechanisms of action of YWLS against HNSCC. The 58 active ingredients from six herbs used for YWLS and their 506 potential targets were screened from the traditional Chinese medicine systems pharmacology database and analysis platform (TCMSP) and SwissTargetPrediction database. A total of 2,173 targets associated with HNSCC were mainly identified from the DisGeNET and GeneCards databases. An active components-targets-disease network was constructed in the Cytoscape. Top 20 hub targets, such as AKT1, EGFR, TNF, ESR1, SRC, HSP90AA1, MAPK3, ERBB2, and CCND1, were identified by a degree in the protein–protein interaction (PPI) network. Gene functional enrichment analysis showed that PI3K-AKT, MAPK, Ras, TNF, and EGFR were the main signaling pathways of YWLS in treating HNSCC. There were 48 intersected targets such as EGFR, AKT1, and TNF that were associated with patients’ outcomes by the univariate Cox analysis, and most of them had increased expression in the tumor as compared to normal tissues. The area under curves of receiver operating characteristic indicated their diagnostic potential. Inhibition of these survival-related targets and/or combination with EGFR or AKT inhibitors were promising therapeutic options in HNSCC. The partial active components of YWLS exhibited good binding with the hub targets, and ADME analysis further evaluated the drug-likeness of the active components. These compounds and targets identified in this study might provide novel treatment strategies for HNSCC patients, and the subsequent work is essential to verify the underlying mechanisms of YWLS against HNSCC.

## Introduction

Head and neck cancer (HNC) is one of the most common and aggressive human malignancies worldwide and is also one of the most lethal causes of death (Johnson et al., 2020). HNC is characterized by the heterogeneity of primary sites where the tumor originates, including the oral cavity, nasopharynx, oropharynx, larynx, tongue, and hypopharynx (Rasmussen et al., 2019). HNC is understood to be primarily comprised of squamous cell carcinoma, accounting for greater than 90% of cases. Genetic heterogeneity, alcohol consumption, and tobacco abuse are considered the leading carcinogens. Infection with human papillomavirus (HPV) (Chaturvedi et al., 2011) and Epstein–Barr virus (Chien et al., 2001) are also known causes of HNC formation. The standard treatments for HNC with advanced stages are surgery, radiation therapy, chemotherapy, and chemoradiotherapy. The advancement in molecular targeted therapy and immunotherapy has provided promising therapeutic options for patients with metastatic or recurrent HNC (Casasola, 2010). However, the poor outcome of these therapies has not been improved in recent years (Siegel et al., 2022). Therefore, the identification of novel prognostic biomarkers or effective therapeutics is an urgent need.

Most patients with head and neck squamous cell carcinoma (HNSCC) are diagnosed at advanced stages and have a 40–50% 5-year survival rate when receiving standard therapies (Gregoire et al., 2010). The survival of recurrent or metastatic HNSCC was even worse with median overall survival (OS) of 1 year (Argiris et al., 2017). Meanwhile, potentially life-threatening complications or side effects caused by most therapies for HNSCC patients, such as swallowing trouble, nerve damage, dry mouth, substantial toxicity, and hearing loss, are big challenges to be solved (Johnson et al., 2020). Clinical studies have shown that traditional Chinese medicine (TCM) was effective in treating HNSCC and its complications, such as *Poria cocos* (PC) and *Atractylodes macrocephala koidz* (AMK) (Meng et al., 2018; Cheng et al., 2021). In addition, it has been reported that *Artemisiae scopariae herba* (ASH) and Wuling San have anti-tumor efficacy (Lu, 2012). For example, they can be used to decrease chemoradiotherapy-induced diarrhea and ascites (Qu et al., 2016). Yinchen Wuling San (YWLS) prescription is a traditional Chinese medicine from Synopsis of Golden Chamber and consists of six herbal materials including *Artemisiae scopariae herba* (ASH, Chinese name: Yinchen), *Poria cocos* (PC, Chinese name: Fuling), *Alisma orientale* (AO, Chinese name: Zexie), *Atractylodes macrocephala koidz* (AMK, Chinese name: Baizhu), *Polyporus umbellatus* (PU, Chinese name: Zhuling), and *Cinnamomi ramulus* (CR, Chinese name: Guizhi) (Yao et al., 2016). Thus, these Chinese herbs might be potential alternatives or complements for HNSCC. However, the biochemical active components and anti-tumor mechanism of YWLS are unclear and need to be explored.

**FIGURE 1:**
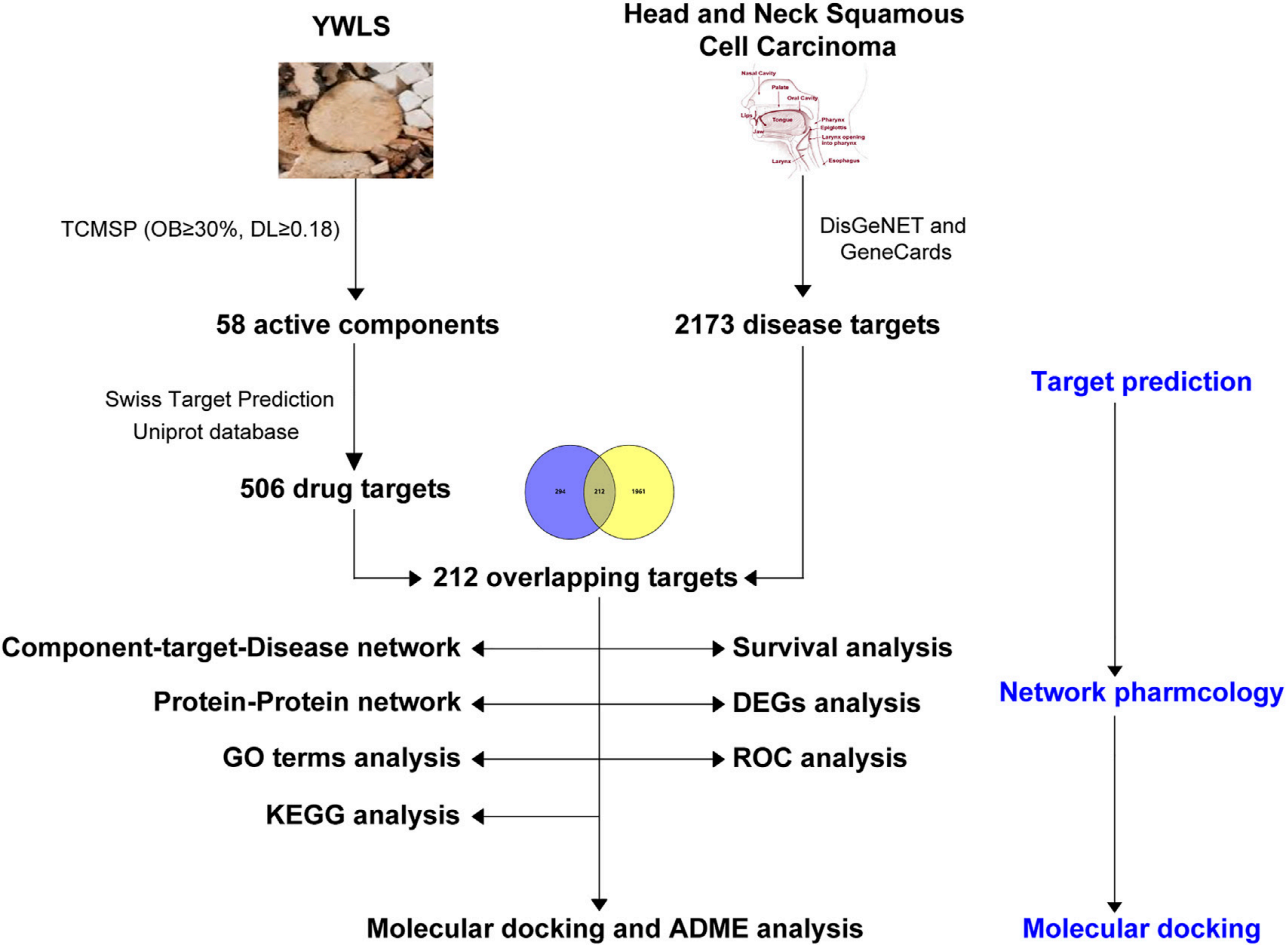
The workflow of this study.

Network pharmacology as a novel analytical approach has been widely used to predict pharmacological action and potential mechanisms of TCM through integrating drug targets, diseases, and their targets into biomolecular networks (Li and Zhang, 2013) (Sun et al., 2021). In this study, network pharmacology was employed to screen active ingredients of YWLS and their potential targets and to explore the action mechanisms of YWLS against HNSCC. Furthermore, the molecular interactions of identified components with their possible targets were predicted by molecular docking studies. The present study might provide underlying mechanisms of YWLS against HNSCC, and the found targets and therapeutic clues are expected to be validated in further experiments. An analysis workflow of this study is illustrated in Figure 1.

## Methods and Materials

### Screening of Active Ingredients of Yinchen Wuling San Prescription

YWLS is a common traditional Chinese prescription that includes six herbs: ASH, PC, AO, AMK, CR, and PU. These herbs contain a variety of compounds with the effects of anti-inflammatory, antioxidant, immune regulation, and anti-tumor. ASH has been demonstrated to induce KB epithelioid cell apoptosis through elevated mitochondrial stress and caspase activation mediated by the MAPK-stimulated signaling pathway (Cha et al., 2009). The active ingredients of these herbs were screened from the TCMSP database (http://lsp.nwu.edu.cn/tcmsp.php) (Ru et al., 2014) with parameters of drug-like properties (DL) ≥ 0.18 and oral bioavailability (OB) ≥ 30%. The available pharmacological targets of these ingredients in each herb were obtained from the SwissTargetPrediction database (http://www.swisstargetprediction.ch/) (Daina et al., 2019) since it covers more targets than the TCMSP database. Additionally, unpredicted known targets for active ingredients were added based on published literature. The UniProt database (https://www.uniprot.org/) was used to standardize gene names and target information.

### Identification of Potential Targets of Head and Neck Squamous Cell Carcinoma

The DisGeNET (Pinero et al., 2015) and GeneCards (Fishilevich et al., 2017) databases were employed to screen pathological targets of HNSCC. The potential HNSCC-related targets were obtained by merging the two database-derived targets after deleting duplicates. In addition, YWLS and HNSCC-related targets were intersected by the Venn diagram.

### Protein–Protein Interaction Network and Topological Analysis

To investigate potential interactions among intersecting targets of YWLS and HNSCC, the PPI network was obtained using the STRING database (Szklarczyk et al., 2021) and visualized in Cytoscape (version 3.8.0) (Shannon et al., 2003). The densely connected modules in the network were identified using the Molecular Complex Detection (MCODE) plugin (Bader and Hogue, 2003) with the default parameters (“Degree Cutoff = 2,” “Node Score Cutoff = 0.2,” “K-Core = 2,” and “Max. Depth = 100.”). The CytoNCA plugin (Tang et al., 2015) was used to calculate the nodes with the highest degree. The hub genes were retrieved by degree using the cytoHubba plugin (Chin et al., 2014).

### GO and Kyoto Encyclopedia of Genes and Genomes Enrichment Analysis

To interrogate the potential functions of these intersecting targets of YWLS and HNSCC, gene functional enrichment analysis including biological process (BP), molecular function (MF), and cellular components (CC) was performed using the clusterProfiler package (Yu et al., 2012). The pathway referenced from the Kyoto Encyclopedia of Genes and Genomes pathways (KEGG) was also investigated. Moreover, among these targets, the KEGG pathways of the targets of each herb and immune-related targets involved were also investigated. Additionally, the important targets that were involved in the significantly enriched pathways were visualized in the pathway maps using the Pathview R package (Luo and Brouwer, 2013).

### Construction of Active Compounds of Yinchen Wuling San Prescription–Head and Neck Squamous Cell Carcinoma Disease Regulatory Network

In order to illustrate the regulatory network of all active compounds of YWLS and their corresponding targets and HNSCC-related targets, the compound–disease regulatory network was generated by Perl and visualized using the Cytoscape software (Shannon et al., 2003).

### Prognostic Effect of Intersecting Targets in Head and Neck Squamous Cell Carcinoma Patients

Gene expression profiles measured by Fragments Per Kilobase of transcript per Million mapped reads (Log2 (FPKM+1)) and clinical information of HNSCC patients were acquired from The Cancer Genome Atlas (TCGA) database (https://portal.gdc.cancer.gov/). The expression matrix of intersecting targets in each HNSCC patient was extracted. The expression levels of these genes between normal and cancerous tissues were compared using Wilcoxon tests and illustrated as a heatmap by the pheatmap R package. To determine their prognostic utility, univariate Cox regression analysis was employed to screen overall survival (OS)-related genes.

An independent HNSCC dataset (GSE42743, n = 103) (Lohavanichbutr et al., 2013) was used to validate the survival-related target expression pattern in tumor and normal tissues.

### Molecular Docking

The overlapped genes of the top 20 hub targets and the genes that have a prognostic effect were searched in the RCSB PDB database (https://www.rcsb.org), and their available 3D protein conformations with resolutions less than 3Å as determined by X-ray crystal diffraction were used. The structures of the selected active ingredients of YWLS were downloaded from the PubChem database (SDF.files). SybylX-2.0 software was used to perform energy minimization and optimize geometry in the Tripos force field. SybylX-2.0 software was applied for molecular docking studies. We processed proteins as follows: removing the co-crystalized ligand and water molecules from the structure, adding the H atoms, and fixing the terminal. The Surflex-Dock (SFXC) docking mode was applied, and the obtained total scores usually indicate the binding force.

It is widely believed that the total score >4.0 indicates that the docking ligands have certain binding activity with the target, the total score >5.0 indicates good binding activity, and the total score >7.0 indicates strong binding activity (Lohning et al., 2017). Meanwhile, export the protein and small-molecule docking file and import the file into PyMOL to visualize the results.

### Absorption, Distribution, Metabolism, and Excretion Analysis of Active Molecules

To evaluate potential active ingredients with good ADME characteristics, pharmacokinetic properties, drug-likeness, and medicinal chemistry friendliness of these molecules were predicted using the SwissADME database (http://www.swissadme.ch/) (Daina et al., 2017).

## Results

### Active Ingredients of Yinchen Wuling San Prescription

A total of 58 active ingredients of YWLS with OB ≥ 30% and DL ≥ 0.18 were acquired in the TCMSP database (Table 1). There were 11 compounds from PU, 10 compounds from AO, 15 compounds from PC, 7 compounds from AMK, 7 compounds from CR, and 13 compounds from ASH, respectively. We noted that CR and AO share the sitosterol ingredient, CR and ASH have the same ingredient beta-sitosterol, and CR and PU share the peroxyergosterol ingredient. Cerevisterol and ergosta-7,22E-dien-3beta-ol were the same compounds from PU and PC.

**TABLE 1:** Active components of YWLS.

Mol ID	Molecule name	OB (%)	DL	Herb name
MOL000359	Sitosterol	36.91	0.75	Alisma orientale
MOL000830	Alisol B	34.47	0.82	Alisma orientale
MOL000831	Alisol B monoacetate	35.58	0.81	Alisma orientale
MOL000832	Alisol,b,23-acetate	32.52	0.82	Alisma orientale
MOL000849	16β-methoxyalisol B monoacetate	32.43	0.77	Alisma orientale
MOL000853	Alisol B	36.76	0.82	Alisma orientale
MOL000854	Alisol C	32.7	0.82	Alisma orientale
MOL000856	Alisol C monoacetate	33.06	0.83	Alisma orientale
MOL002464	1-monolinolein	37.18	0.3	Alisma orientale
MOL000862	Alisol B 23-acetate	35.58	0.81	Alisma orientale
MOL000279	Cerevisterol	37.96	0.77	Polyporus umbellatus
MOL000282	Ergosta-7,22E-dien-3beta-ol	43.51	0.72	Polyporus umbellatus
MOL000796	(22e,24r)-ergosta-6-en-3beta,5alpha,6beta-triol	30.2	0.76	Polyporus umbellatus
MOL000797	(22e,24r)-ergosta-7,22-dien-3-one	44.88	0.72	Polyporus umbellatus
MOL000798	Ergosta-7,22-diene-3β-ol	43.51	0.72	Polyporus umbellatus
MOL000801	5alpha,8alpha-epidioxy-(22e,24r)-ergosta-6,22-dien-3beta-ol	44.39	0.82	Polyporus umbellatus
MOL011169	Peroxyergosterol	44.39	0.82	Polyporus umbellatus
MOL000816	Ergosta-7,22-dien-3-one	44.88	0.72	Polyporus umbellatus
MOL000817	Ergosta-5,7,22-trien-3-ol	46.18	0.72	Polyporus umbellatus
MOL000820	Polyporusterone E	45.71	0.85	Polyporus umbellatus
MOL000822	Polyporusterone G	33.43	0.81	Polyporus umbellatus
MOL000273	(2R)-2-[(3S,5R,10S,13,14,16,17R)-3,16-dihydroxy-4,4,10,13,14-pentamethyl-2,3,5,6,12.15,16,17-octahydro-1H-cyclopenta [a]phenanthren-17-yl]-6-methylhept-5-enoic acid	30.93	0.81	Poria cocos
MOL000275	Trametenolic acid	38.71	0.8	Poria cocos
MOL000276	7.9 (11)-dehydropachymic acid	35.11	0.81	Poria cocos
MOL000279	Cerevisterol	37.96	0.77	Poria cocos
MOL000280	(2R)-2-[(3S,5R,10S,13,14,16,17R)-3,16-dihydroxy-4,4,10,13,14-pentamethyl-2,3,5,6,12.15,16,17-octahydro-1H-cyclopenta [a]phenanthren-17-yl]-5-isopropyl-hex-5-enoic acid	31.07	0.82	Poria cocos
MOL000282	Ergosta-7,22E-dien-3beta-ol	43.51	0.72	Poria cocos
MOL000283	Ergosterol peroxide	40.36	0.81	Poria cocos
MOL000285	Polyporenic acid C	38.26	0.82	Poria cocos
MOL000287	3beta-hydroxy-24-methylene-8-lanostene-21-oic acid	38.7	0.81	Poria cocos
MOL000289	Pachymic acid	33.63	0.81	Poria cocos
MOL000290	Poricoic acid A	30.61	0.76	Poria cocos
MOL000291	Poricoic acid B	30.52	0.75	Poria cocos
MOL000292	Poricoic acid C	38.15	0.75	Poria cocos
MOL000296	Hederagenin	36.91	0.75	Poria cocos
MOL000300	Dehydroeburicoic acid	44.17	0.83	Poria cocos
MOL000072	8β-ethoxy atractylenolide Ⅲ	35.95	0.21	Atractylodes macrocephala koidz
MOL000033	(3,8,9S,10R,13R,14S,17R)-10,13-dimethyl-17-[(2R,5S)-5-propan-2-yloctan-2-yl]-2,3,4,7,8,9,11,12,14.15,16,17-dodecahydro-1H-cyclopenta [a]phenanthren-3-ol	36.23	0.78	Atractylodes macrocephala koidz
MOL000028	α-amyrin	39.51	0.76	Atractylodes macrocephala koidz
MOL000049	3β-acetoxyatractylone	54.07	0.22	Atractylodes macrocephala koidz
MOL000021	14-acetyl-12-senecioyl-2E,8E,10E-atractylentriol	60.31	0.31	Atractylodes macrocephala koidz
MOL000020	12-senecioyl-2E,8E,10E-atractylentriol	62.4	0.22	Atractylodes macrocephala koidz
MOL000022	14-acetyl-12-senecioyl-2E,8Z,10E-atractylentriol	63.37	0.3	Atractylodes macrocephala koidz
MOL001736	(-)-taxifolin	60.51	0.27	Cinnamomi ramulus
MOL000358	Beta-sitosterol	36.91	0.75	Cinnamomi ramulus
MOL000359	Sitosterol	36.91	0.75	Cinnamomi ramulus
MOL000492	(+)-catechin	54.83	0.24	Cinnamomi ramulus
MOL000073	Ent-epicatechin	48.96	0.24	Cinnamomi ramulus
MOL004576	Taxifolin	57.84	0.27	Cinnamomi ramulus
MOL011169	Peroxyergosterol	44.39	0.82	Cinnamomi ramulus
MOL000354	Isorhamnetin	49.6	0.31	Artemisiae scopariae herba
MOL000358	Beta-sitosterol	36.91	0.75	Artemisiae scopariae herba
MOL004609	Areapillin	48.96	0.41	Artemisiae scopariae herba
MOL005573	Genkwanin	37.13	0.24	Artemisiae scopariae herba
MOL007274	Skrofulein	30.35	0.3	Artemisiae scopariae herba
MOL008039	Isoarcapillin	57.4	0.41	Artemisiae scopariae herba
MOL008040	Eupalitin	46.11	0.33	Artemisiae scopariae herba
MOL008041	Eupatolitin	42.55	0.37	Artemisiae scopariae herba
MOL008043	Capillarisin	57.56	0.31	Artemisiae scopariae herba
MOL008045	4′-methylcapillarisin	72.18	0.35	Artemisiae scopariae herba
MOL008046	Demethoxycapillarisin	52.33	0.25	Artemisiae scopariae herba
MOL008047	Artepillin A	68.32	0.24	Artemisiae scopariae herba
MOL000098	Quercetin	46.43	0.28	Artemisiae scopariae herba

### Target Prediction of Active Ingredients of Yinchen Wuling San Prescription

The targets of these active ingredients in YWLS were predicted in the SwissTargetPrediction database, and 506 potential targets were obtained after the duplicate deletion (Supplementary Table S1). Among these targets, a total of 131 targets are immune-related genes, which are mainly categorized into cytokines and their receptors, BCR signaling pathway, antimicrobials, natural killer cell cytotoxicity, and TCR signaling pathway, suggesting that active components of YWLS might act through modulating immune response (Supplementary Table S1). Furthermore, the most enriched GO terms and KEGG pathways of these immune-related genes are the same. Some signaling pathways related to immune regulation including T cell receptor, VEGF, and Fc epsilon RI signaling corroborated the conjecture (Supplementary Figure S1A, B).

### Disease-Related Targets Prediction of Head and Neck Squamous Cell Carcinoma

**FIGURE 2:**
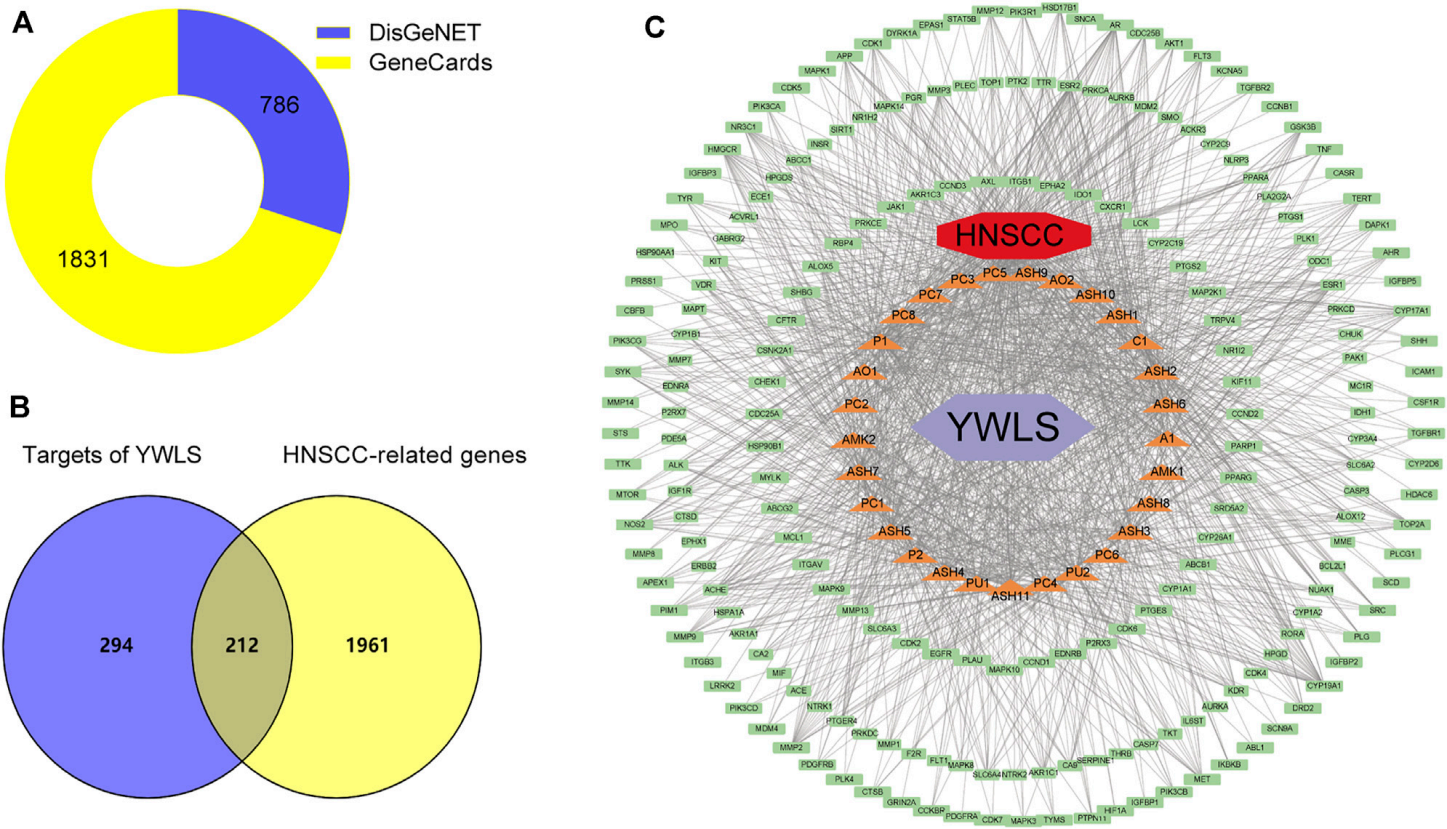
Construction of compound-target-disease network. **(A)**. Screening of head and neck squamous cell carcinoma related targets from DisGeNET and GeneCards databases. **(B)** The Venny plot of 212 potential targets. **(C)** The active component-target-disease network. The red diamond represented the disease; the light green rectangle represented intersecting targets; the light purple diamond represented YWLS; the orange triangle represented active compounds (C1, MOL000359; AO1, MOL002464; AO2, MOL000862; P1, MOL000279; P2, MOL000282; PU1, MOL000796; PU2, MOL000820; PC1, MOL000273; PC2, MOL000276; PC3, MOL000280; PC4, MOL000285, PC5, MOL000287; PC6, MOL000290; PC7, MOL000291; PC8, MOL000292; AMK1, MOL000072; AMK2, MOL000033; A1, MOL000358; ASH1, MOL000354; ASH2, MOL004609; ASH3, MOL005573; ASH4, MOL007274; ASH5, MOL008039; ASH6, MOL008040; ASH7, MOL008041; ASH8, MOL008043; ASH9, MOL008045; ASH10, MOL008046; and ASH11, MOL000098). The edges represented the connection among active components, targets, and disease.

The keywords “head and neck carcinoma” and “head and neck squamous cell carcinoma” were used to search in DisGeNET and GeneCards databases. A total of 2,173 potential pathological targets related to HNSCC were acquired (Figure 2A, Supplementary Table S2). The related targets of HNSCC and active ingredients of YWLS were intersected using the Venn diagram, and 212 disease- and ingredient-related targets were obtained (Figure 2B). The genes corresponding to these targets were further confirmed by the UniProt database (Table 2).

**TABLE 2:** The 212 intersecting potential targets of HNSCC and YWLS.

Gene symbol	Uniprot ID	ChEMBL ID	Target class
HMGCR	P04035	CHEMBL402	Oxidoreductase
AR	P10275	CHEMBL1871	Nuclear receptor
CYP17A1	P05093	CHEMBL3522	Cytochrome P450
CYP19A1	P11511	CHEMBL1978	Cytochrome P450
ESR2	Q92731	CHEMBL242	Nuclear receptor
ESR1	P03372	CHEMBL206	Nuclear receptor
SHBG	P04278	CHEMBL3305	Secreted protein
CYP2C19	P33261	CHEMBL3622	Cytochrome P450
SLC6A2	P23975	CHEMBL222	Electrochemical transporter
RORA	P35398	CHEMBL5868	Nuclear receptor
SLC6A4	P31645	CHEMBL228	Electrochemical transporter
ACHE	P22303	CHEMBL220	Hydrolase
VDR	P11473	CHEMBL1977	Nuclear receptor
NR1H2	P55055	CHEMBL4093	Nuclear receptor
CDC25A	P30304	CHEMBL3775	Phosphatase
NOS2	P35228	CHEMBL4481	Enzyme
NR3C1	P04150	CHEMBL2034	Nuclear receptor
CDC25B	P30305	CHEMBL4804	Phosphatase
SHH	Q15465	CHEMBL5602	Unclassified protein
DRD2	P14416	CHEMBL217	Family A G protein-coupled receptor
PRKCA	P17252	CHEMBL299	Kinase
PRKCD	Q05655	CHEMBL2996	Kinase
ALOX5	P09917	CHEMBL215	Oxidoreductase
PTGS2	P35354	CHEMBL230	Oxidoreductase
PTGES	O14684	CHEMBL5658	Enzyme
HPGD	P15428	CHEMBL1293255	Enzyme
MAPK8	P45983	CHEMBL2276	Kinase
ERBB2	P04626	CHEMBL1824	Kinase
EGFR	P00533	CHEMBL203	Kinase
MAPK14	Q16539	CHEMBL260	Kinase
CHEK1	O14757	CHEMBL4630	Kinase
MTOR	P42345	CHEMBL2842	Kinase
PIK3CA	P42336	CHEMBL4005	Enzyme
AKT1	P31749	CHEMBL4282	Kinase
ICAM1	P05362	CHEMBL2096661	Membrane receptor
SCN9A	Q15858	CHEMBL4296	Voltage-gated ion channel
AURKB	Q96GD4	CHEMBL2185	Kinase
CHUK	O15111	CHEMBL3476	Kinase
RBP4	P02753	CHEMBL3100	Secreted protein
MAP2K1	Q02750	CHEMBL3587	Kinase
CDK6	Q00534	CHEMBL2508	Kinase
CDK4	P11802	CHEMBL331	Kinase
FLT1	P17948	CHEMBL1868	Kinase
EPHX1	P07099	CHEMBL1968	Protease
CDK2	P24941	CHEMBL301	Kinase
CDK7	P50613	CHEMBL3055	Kinase
PLK4	O00444	CHEMBL3788	Kinase
TTK	P33981	CHEMBL3983	Kinase
CDK5	Q00535	CHEMBL4036	Kinase
FLT3	P36888	CHEMBL1974	Kinase
CSF1R	P07333	CHEMBL1844	Kinase
ABL1	P00519	CHEMBL1862	Kinase
MDM2	Q00987	CHEMBL5023	Other nuclear protein
PAK1	Q13153	CHEMBL4600	Kinase
PRKCE	Q02156	CHEMBL3582	Kinase
TRPV4	Q9HBA0	CHEMBL3119	Voltage-gated ion channel
P2RX3	P56373	CHEMBL2998	Ligand-gated ion channel
KDR	P35968	CHEMBL279	Kinase
F2R	P25116	CHEMBL3974	Family A G protein-coupled receptor
CTSD	P07339	CHEMBL2581	Protease
SLC6A3	Q01959	CHEMBL238	Electrochemical transporter
PDGFRA	P16234	CHEMBL2095189	Kinase
PDGFRB	P09619	CHEMBL2095189	Kinase
STS	P08842	CHEMBL3559	Enzyme
MAPK1	P28482	CHEMBL4040	Kinase
KCNA5	P22460	CHEMBL4306	Voltage-gated ion channel
F2	P00734	CHEMBL204	Protease
IKBKB	O14920	CHEMBL1991	Kinase
SMO	Q99835	CHEMBL5971	Frizzled family G protein-coupled receptor
MC1R	Q01726	CHEMBL3795	Family A G protein-coupled receptor
JAK1	P23458	CHEMBL2835	Kinase
NR1I2	O75469	CHEMBL3401	Nuclear receptor
EDNRB	P24530	CHEMBL1785	Family A G protein-coupled receptor
AURKA	O14965	CHEMBL4722	Kinase
MMP1	P03956	CHEMBL332	Protease
IGF1R	P08069	CHEMBL1957	Kinase
MMP13	P45452	CHEMBL280	Protease
CCKBR	P32239	CHEMBL298	Family A G protein-coupled receptor
MMP2	P08253	CHEMBL333	Protease
MMP14	P50281	CHEMBL3869	Protease
APP	P05067	CHEMBL2487	Membrane receptor
TGFBR2	P37173	CHEMBL4267	Kinase
TGFBR1	P36897	CHEMBL4439	Kinase
MET	P08581	CHEMBL3717	Kinase
ACKR3	P25106	CHEMBL2010631	Family A G protein-coupled receptor
IDH1	O75874	CHEMBL2007625	Enzyme
AKR1C3	P42330	CHEMBL4681	Enzyme
KIF11	P52732	CHEMBL4581	Other cytosolic protein
CCND1	P24385	CHEMBL1907601	Kinase
IL6ST	P40189	CHEMBL3124734	Membrane receptor
PRKDC	P78527	CHEMBL3142	Kinase
ALK	Q9UM73	CHEMBL4247	Kinase
KIT	P10721	CHEMBL1936	Kinase
GRIN2A	Q12879	CHEMBL1907604	Ligand-gated ion channel
MDM4	O15151	CHEMBL1255126	Unclassified protein
SYK	P43405	CHEMBL2599	Kinase
CDK1	P06493	CHEMBL1907602	Other cytosolic protein
CCNB1	P14635	CHEMBL1907602	Other cytosolic protein
CYP2D6	P10635	CHEMBL289	Cytochrome P450
PIK3CB	P42338	CHEMBL3145	Enzyme
CYP2C9	P11712	CHEMBL3397	Cytochrome P450
CYP3A4	P08684	CHEMBL340	Cytochrome P450
CCND3	P30281	CHEMBL2095942	Other cytosolic protein
CCND2	P30279	CHEMBL2095942	Other cytosolic protein
MAPK10	P53779	CHEMBL2637	Kinase
TKT	P29401	CHEMBL4983	Enzyme
PTGER4	P35408	CHEMBL1836	Family A G protein-coupled receptor
PDE5A	O76074	CHEMBL1827	Phosphodiesterase
GABRG2	P18507	CHEMBL2094120	Ligand-gated ion channel
CTSB	P07858	CHEMBL4072	Protease
PIK3CD	O00329	CHEMBL3130	Enzyme
PIK3CG	P48736	CHEMBL3267	Enzyme
DYRK1A	Q13627	CHEMBL2292	Kinase
GSK3B	P49841	CHEMBL262	Kinase
HDAC6	Q9UBN7	CHEMBL1865	Eraser
PGR	P06401	CHEMBL208	Nuclear receptor
NLRP3	Q96P20	CHEMBL1741208	Unclassified protein
HIF1A	Q16665	CHEMBL4261	Transcription factor
AXL	P30530	CHEMBL4895	Kinase
PARP1	P09874	CHEMBL3105	Enzyme
CASP3	P42574	CHEMBL2334	Protease
CASP7	P55210	CHEMBL3468	Protease
NTRK1	P04629	CHEMBL2815	Kinase
P2RX7	Q99572	CHEMBL4805	Ligand-gated ion channel
ACVRL1	P37023	CHEMBL5311	Kinase
MAPK9	P45984	CHEMBL4179	Kinase
LRRK2	Q5S007	CHEMBL1075104	Kinase
CBFB	Q13951	CHEMBL1615386	Unclassified protein
EPAS1	Q99814	CHEMBL1744522	Unclassified protein
TNF	P01375	CHEMBL1825	Secreted protein
TOP2A	P11388	CHEMBL1806	Isomerase
MMP3	P08254	CHEMBL283	Protease
PPARA	Q07869	CHEMBL239	Nuclear receptor
PTPN11	Q06124	CHEMBL3864	Phosphatase
ITGB1	P05556	CHEMBL1907599	Membrane receptor
PPARG	P37231	CHEMBL235	Nuclear receptor
ALOX12	P18054	CHEMBL3687	Enzyme
THRB	P10828	CHEMBL1947	Nuclear receptor
ACE	P12821	CHEMBL1808	Protease
EDNRA	P25101	CHEMBL252	Family A G protein-coupled receptor
ECE1	P42892	CHEMBL4791	Protease
ITGAV	P06756	CHEMBL1907598	Membrane receptor
ITGB3	P05106	CHEMBL1907598	Membrane receptor
PRSS1	P07477	CHEMBL209	Protease
STAT5B	P51692	CHEMBL5817	Transcription factor
CASR	P41180	CHEMBL1878	Family C G protein-coupled receptor
PLCG1	P19174	CHEMBL3964	Enzyme
PLEC	Q15149	CHEMBL1293240	Unclassified protein
PLA2G2A	P14555	CHEMBL3474	Enzyme
TYMS	P04818	CHEMBL1952	Transferase
EPHA2	P29317	CHEMBL2068	Kinase
SRD5A2	P31213	CHEMBL1856	Oxidoreductase
MME	P08473	CHEMBL1944	Protease
SERPINE1	P05121	CHEMBL3475	Secreted protein
MIF	P14174	CHEMBL2085	Enzyme
MMP7	P09237	CHEMBL4073	Protease
HPGDS	O60760	CHEMBL5879	Transferase
MCL1	Q07820	CHEMBL4361	Other cytosolic protein
MMP9	P14780	CHEMBL321	Protease
HSP90AA1	P07900	CHEMBL3880	Other cytosolic protein
MMP12	P39900	CHEMBL4393	Protease
TERT	O14746	CHEMBL2916	Enzyme
SCD	O00767	CHEMBL5555	Enzyme
TOP1	P11387	CHEMBL1781	Isomerase
PTGS1	P23219	CHEMBL221	Oxidoreductase
MAPK3	P27361	CHEMBL3385	Kinase
IDO1	P14902	CHEMBL4685	Enzyme
CYP26A1	O43174	CHEMBL5141	Cytochrome P450
BCL2L1	Q07817	CHEMBL4625	Other ion channel
CA9	Q16790	CHEMBL3594	Lyase
CA2	P00918	CHEMBL205	Lyase
CYP1B1	Q16678	CHEMBL4878	Cytochrome P450
ABCC1	P33527	CHEMBL3004	Primary active transporter
ABCG2	Q9UNQ0	CHEMBL5393	Primary active transporter
PIM1	P11309	CHEMBL2147	Kinase
MPO	P05164	CHEMBL2439	Enzyme
PIK3R1	P27986	CHEMBL2506	Enzyme
DAPK1	P53355	CHEMBL2558	Kinase
SRC	P12931	CHEMBL267	Kinase
PTK2	Q05397	CHEMBL2695	Kinase
PLK1	P53350	CHEMBL3024	Kinase
CSNK2A1	P68400	CHEMBL3629	Kinase
CXCR1	P25024	CHEMBL4029	Family A G protein-coupled receptor
ABCB1	P08183	CHEMBL4302	Primary active transporter
NUAK1	O60285	CHEMBL5784	Kinase
AKR1C1	Q04828	CHEMBL5905	Enzyme
AKR1A1	P14550	CHEMBL2246	Enzyme
MAPT	P10636	CHEMBL1293224	Unclassified protein
INSR	P06213	CHEMBL1981	Kinase
MYLK	Q15746	CHEMBL2428	Kinase
APEX1	P27695	CHEMBL5619	Enzyme
TYR	P14679	CHEMBL1973	Oxidoreductase
HSD17B1	P14061	CHEMBL3181	Enzyme
AHR	P35869	CHEMBL3201	Transcription factor
PLG	P00747	CHEMBL1801	Protease
TTR	P02766	CHEMBL3194	Secreted protein
ODC1	P11926	CHEMBL1869	Lyase
CFTR	P13569	CHEMBL4051	Other ion channel
LCK	P06239	CHEMBL258	Kinase
CYP1A1	P04798	CHEMBL2231	Cytochrome P450
CYP1A2	P05177	CHEMBL3356	Cytochrome P450
NTRK2	Q16620	CHEMBL4898	Kinase
HSPA1A	P0DMV8	CHEMBL5460	Other cytosolic protein
PLAU	P00749	CHEMBL3286	Protease
SIRT1	Q96EB6	CHEMBL4506	Eraser
HSP90B1	P14625	CHEMBL1075323	Other membrane protein
MMP8	P22894	CHEMBL4588	Protease
IGFBP3	P17936	CHEMBL3997	Secreted protein
SNCA	P37840	CHEMBL6152	Unclassified protein
IGFBP5	P24593	CHEMBL2665	Secreted protein
IGFBP2	P18065	CHEMBL3088	Secreted protein
IGFBP1	P08833	CHEMBL4178	Secreted protein

### Construction of the Compound–Disease Regulatory Network

The ingredient-target-disease interaction network was established using Perl and constructed via Cytoscape (Figure 2C), and 242 nodes and 2,640 edges constituted the network. Active compound cerevisterol had the most nodes. (22e,24r)-ergosta-6-en-3beta,5alpha,6beta-triol and polyporusterone E ranked as the secondary and tertiary central nodes, respectively, suggesting they might be the most efficacious components against HNSCC with multiple effects by interacting with different targets (Supplementary Figure S1C, Supplementary Table S3).

### PPI Network Analysis

**FIGURE 3:**
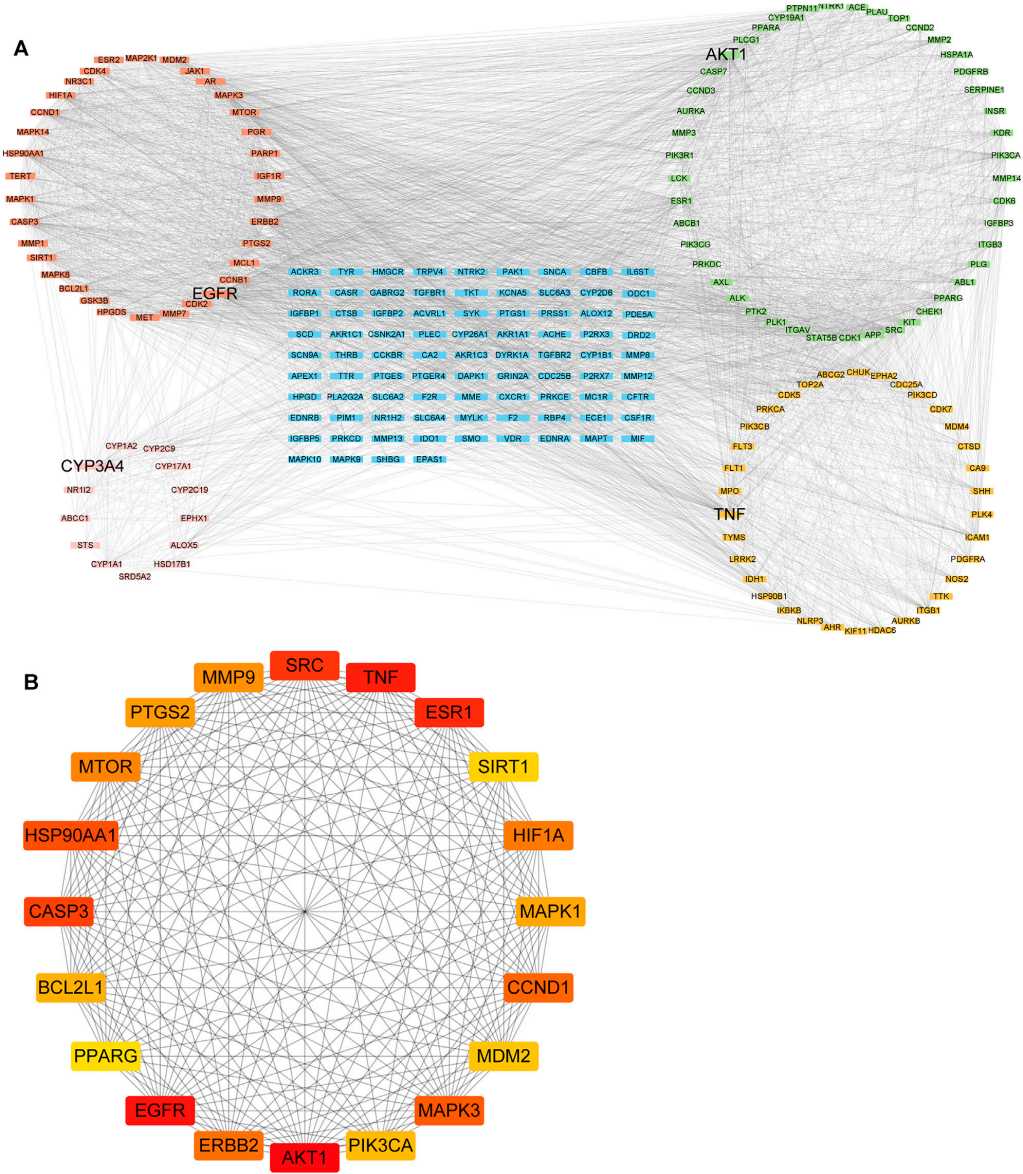
The protein–protein network of 212 intersecting targets. **(A)** Four modules (EGFR, AKT1, TNF, and CYP3A4) were identified from the whole PPI network. **(B)** The top 20 Core targets are determined by the degree. Color represented the target degree.

The PPI analysis was performed to investigate the potential interactions of 212 targets. Four significant modules, AKT1, EGFR, TNF, and CYP3A4, were identified in the whole network (Figure 3A). The AKT1 module contained 45 nodes and 342 edges, the EGFR module had 34 nodes and 411 edges, and the TNF module comprised 34 nodes and 97 edges. CYP3A4, CYP2C9, and CYP1A1 were the top three nodes of the CYP3A4 module, which belong to the most common drug-metabolizing enzymes (DME) that contribute significantly to the elimination pathways of new chemical entities (Di, 2014). Furthermore, the top 20 targets ranked by degree in the network were regarded as the hub genes (Figure 3B). Among them, AKT1, EGFR, and TNF were the top 3 hub genes according to the degree. These hub genes might have important implications for the pathogenesis of HNSCC. AKT1 can restrict the invasive capacity of HNC cells through the EGFR-PI3K-AKT-mTOR signaling axis (Brolih et al., 2018) and was involved in acquired cetuximab resistant HNSCC (Zaryouh et al., 2021). Meanwhile, EGFR has been reported as anti-tumor target due to its important role in cell proliferation and survival (Burtness, 2005). Moreover, TNF signaling plays a tumor-promoting role by inducing suppressive tumor immune microenvironment and apoptosis resistance in HNSCC (Sandra et al., 2002; Jackson-Bernitsas et al., 2007; Lu et al., 2011). Blockades of these targets represent potential therapeutics for tumors including HNSCC.

### Functional Enrichment Analysis

**FIGURE 4:**
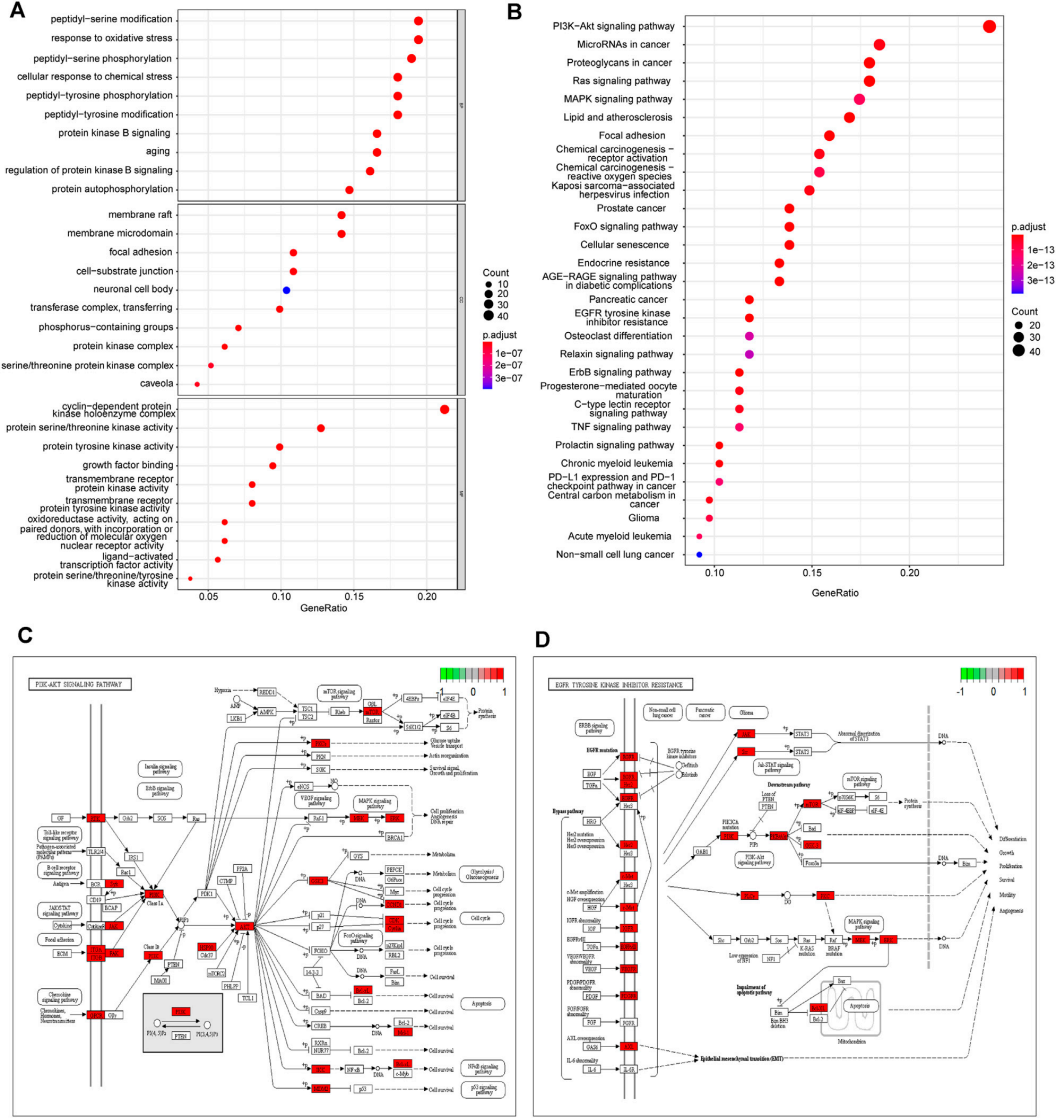
GO and KEGG enrichment analysis. **(A)** The top 10 enriched GO items of BP, CC, and MF. **(B)** The top 30 enriched KEGG pathways. **(C)** The important target genes were mainly distributed in the PI3K-AKT pathway. **(D)** The important target genes were mainly distributed in the EGFR tyrosine kinase inhibitor resistance pathway.

To investigate the biological functions of 212 potential targets, GO terms analysis showed that they were mainly involved in the biological processes of response to oxidative and chemical stress, peptidyl-serine/tyrosine modification, and protein kinase B signaling pathway. Membrane raft and microdomain, focal adhesion and cell-substrate junction, and protein kinase complex were the main cellular components. Protein kinase activity, growth factor binding activity, and nuclear receptor and ligand-activated transcription factor activity are the top molecular functions (Figure 4A). The pathways referenced from the KEGG database indicated that these targets were enriched in various signaling pathways related to human malignancies, including PI3K-AKT, Ras signaling, MAPK signaling, chemical carcinogenesis, EGFR tyrosine kinase inhibitor resistance, ErbB signaling, and FoxO signaling pathways (Figure 4B). In addition, most of the KEGG pathways that the targets of each herb from YWLS were involved in the similar pathways (Supplementary Table S4). The targets involved in PI3K-AKT and EGFR tyrosine kinase inhibitor resistance were mapped in the pathway (Figures 4C, D). EGFR-targeting inhibitors, such as cetuximab, have been used to treat HNSCC, however, only a small subset of patients showed responsiveness. This might imply that the targets of active ingredients in YWLS are involved in drug resistance (Grandis et al., 1997).

### Correlation of Intersected Target Expression With Patients’ Overall Survival

**FIGURE 5:**
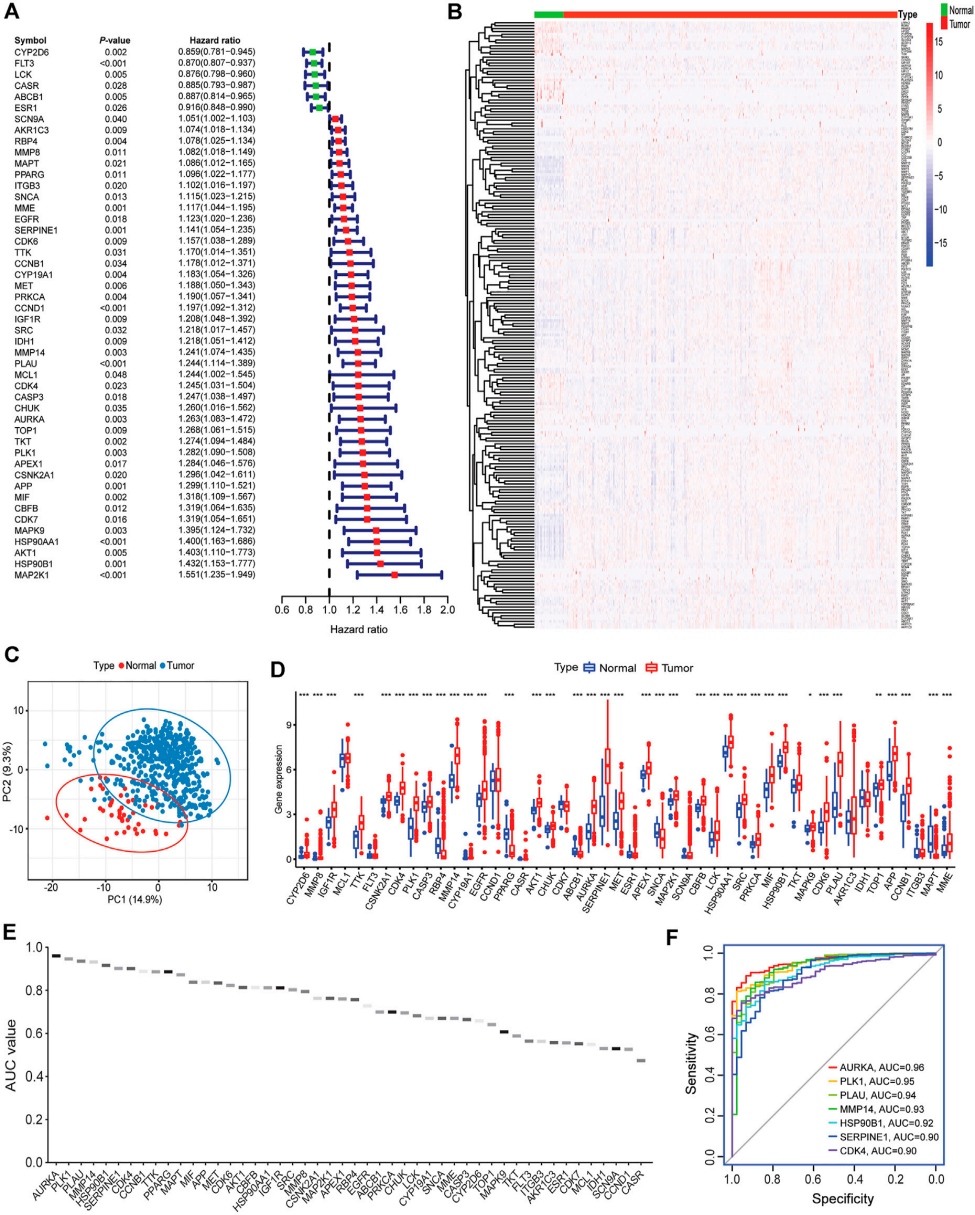
Associations of intersecting targets with patients’ outcomes, normal and tumor samples. **(A)** The forest plot represented the associations of intersecting targets with patients’ outcomes. **(B)** Heatmap of intersecting targets expression in normal vs. tumor samples. **(C)** Principal component analysis showed a distinct expression pattern of intersecting targets in normal vs. tumor samples. **(D)** Expression of 48 survival-related targets in normal vs. tumor samples (Wilcox test, ***: <0.001; **: <0.01; and *: <0.05). **(E)** AUC values of ROC analysis for 48 survival-related targets. **(F)** The top 7 targets that AUC values greater than 0.9.

To determine the clinical relevance of 212 intersecting targets in HNSCC patients, the univariate Cox regression analysis showed that 48 targets were significantly correlated with patients’ outcomes (Figure 5A). Among these survival-related targets, high expression of CYP2D6, FLT3, LCK, CASR, ABCB1, and ESR1 were linked to better survival, suggesting they might act as protective factors, whereas increased expression of the other 42 targets were associated with unfavorable prognosis, indicating they might be risk genes. As for the top 20 hub genes, 7 genes were found to be related to decreased survival in HNSCC patients. For example, patients with high AKT1 and EGFR expression had decreased survival, which was consistent with a previous report (Burtness, 2005).

The principal component analysis (PCA) showed that the expression pattern of 212 intersecting genes in the normal tissues was distinct from those in tumor samples (Figure 5B). As illustrated in Figure 5C, most of these genes had increased expression in tumor tissues than in normal tissues and showed evident expression pattern. In addition, 33 survival-related genes were observed to be elevated in tumor tissues, whereas 4 genes (RBP4, ABCB1, SCNA, and MAPT) showed increased expression in normal tissues (Figure 5D). The distinct expression pattern of survival-related targets was verified in an independent HNSCC cohort (GSE42743, Supplementary Figure S2A), and most of these targets were increased in tumors as compared to normal tissues (Supplementary Figure S2B). Receiver operating characteristic (ROC) was performed to further evaluate the diagnostic capacity of these 48 genes in separating normal from tumor samples. The area under the curve (AUC) of ROC ranged from 0.47 to 0.96 (Figure 5E). The top 7 AUCs >0.9 (AURKA, PLK1, PLAU, MMP14, HSP90B1, SERPINE1, and CDK4) are visualized in Figure 5F, indicating they may be promising targets for anti-HNSCC therapy.

### Molecular Docking

**FIGURE 6:**
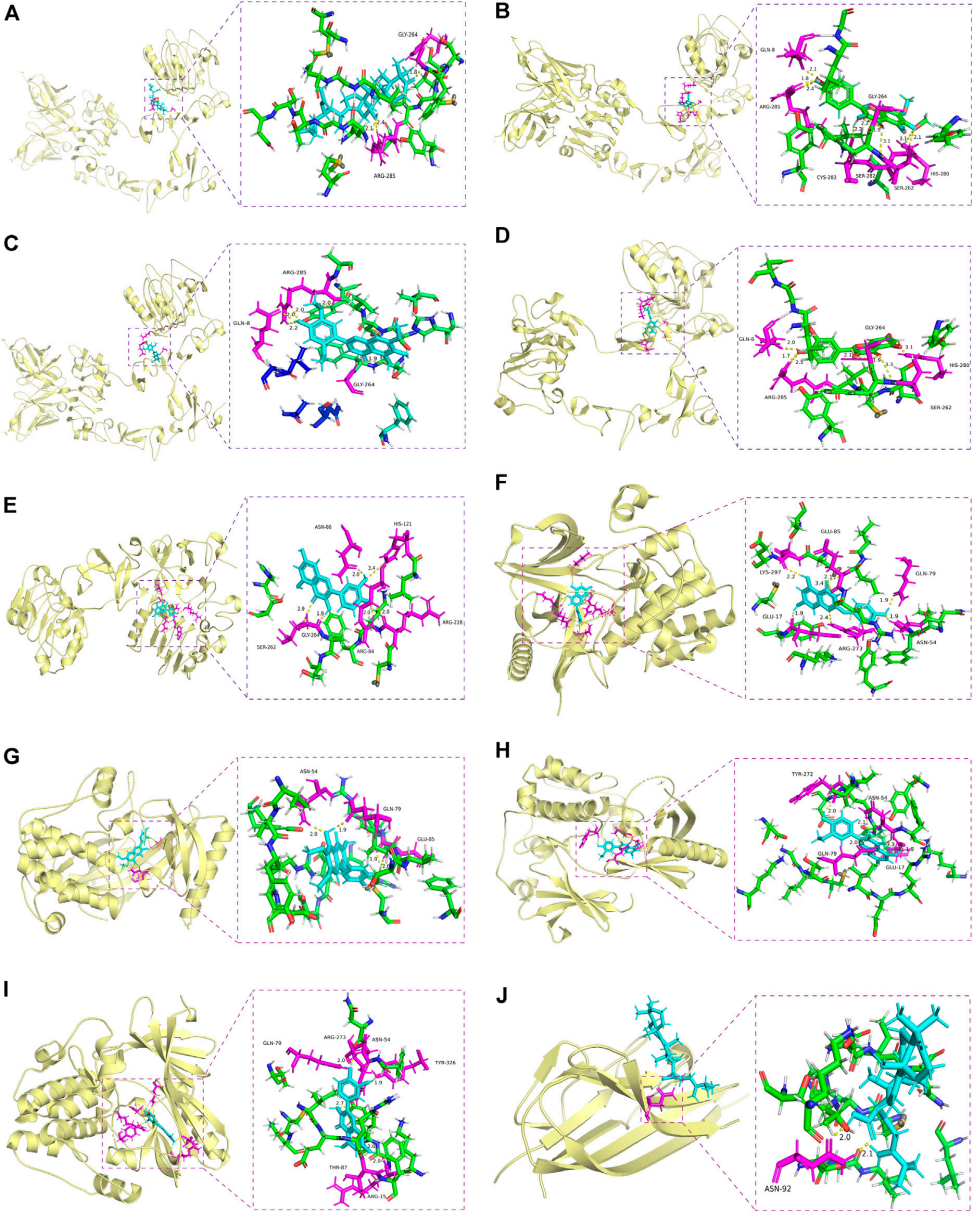
Molecular docking of active compounds in core targets. **(A)** Alisol B 23-acetate-EGFR. **(B)** Eupalitin-EGFR. **(C)** Isoarcapillin-EGFR. **(D)** Isorhamnetin-EGFR. **(E)** Quercetin-EGFR. **(F)** Demethoxycapillarisin-AKT1. **(G)** Eupatolitin-AKT1. **(H)** Isorhamnetin and AKT1. **(I)** Quercetin-AKT1. **(J)** Polyporenic acid **C**-TNF.

Molecular docking is a powerful structure-based approach to characterize the binding behavior of small molecules in the target proteins and elucidate fundamental interactions at the atomic level (Meng et al., 2011). We found that 8 genes were overlapped between 20 hub genes and 48 survival-related genes, including AKT1, EGFR, PPARG, CCND1, SRC, CASP3, HSP90AA1, and ESR1. EGFR inhibitors including gefitinib, erlotinib, and lapatinib have shown limited therapeutic efficacy for HNSCC patients due to tumor resistance (Cohen et al., 2003; Soulieres et al., 2004). Inhibition of AKT1/2/3 with cetuximab has been reported as a promising therapeutic strategy for acquired cetuximab resistance in HNSCC patients (Zaryouh et al., 2021). Activation of SRC, one of the non-receptor tyrosine kinase protein family, promotes cell survival, proliferation, and invasion in various human malignancies including lung, colon, and prostate cancer (Dehm and Bonham, 2004). Several SRC-targeting inhibitors have been in clinical trial phases. For instance, dasatinib was approved to treat chronic myeloid leukemia (Breccia et al., 2013), whereas SRC-based therapy for HNSCC is limited (Lang et al., 2018). Overlapped genes were selected to complete molecular docking with their predicted 11 ingredients of YWLS. Among these ingredients, 10 ingredients had comparable binding scores with the selected target proteins excluding MOL000279 (Table 3 and Figures 6A–J). The docking score of MOL000862 with EGFR was 7.10, suggesting this molecule might interact well with the EGFR protein. Molecular dockings of MOL000285 and MOL005573 in PPARG and MOL008039 and MOL000796 in ESR1 also exhibited high performance. A similar high predicted binding potential was seen in AKT1 with MOL000354, MOL008041, MOL000098, and MOL008046. SRC protein with MOL000354, MOL008040, and MOL008041 showed high binding capacity. The data implied that these compounds might be potential drugs for HNSCC.

**TABLE 3:** Molecular docking of active components with their related targets.

Target name	PDB ID	Mol ID	Mol name	Total score
EGFR	5xwd	MOL000862	Alisol B 23-acetate	7.1
		MOL000354	Isorhamnetin	4.0
		MOL005573	Genkwanin	3.3
		MOL008039	Isoarcapillin	4.2
		MOL008040	Eupalitin	4.2
		MOL008041	Eupatolitin	3.7
		MOL000098	Quercetin	4.0
AKT1	6hhg	MOL000354	Isorhamnetin	5.4
		MOL008041	Eupatolitin	6.6
		MOL000098	Quercetin	6.2
		MOL008046	Demethoxycapillarisin	6.9
SRC	2h8h	MOL000354	Isorhamnetin	5.6
		MOL005573	Genkwanin	4.6
		MOL008039	Isoarcapillin	4.5
		MOL008040	Eupalitin	5.9
		MOL008041	Eupatolitin	6.8
		MOL000098	Quercetin	4.2
		MOL008046	Demethoxycapillarisin	4.4
ESR1	2ocf	MOL005573	Genkwanin	4.5
		MOL008039	Isoarcapillin	5.6
		MOL000285	Polyporenic acid C	4.5
		MOL000279	Cerevisterol	3.6
		MOL000796	(22e,24r)-ergosta-6-en-3beta,5alpha,6beta-triol	6.1
PPARG	3e00	MOL005573	Genkwanin	5.4
		MOL000285	Polyporenic acid C	6.4
HSP90AA1	6gqs	MOL000285	Polyporenic acid C	3.2
TNF	5uui	MOL000285	Polyporenic acid C	4.6
BCL2L1	7jgw	MOL008046	Demethoxycapillarisin	5.4

### Absorption, Distribution, Metabolism, and Excretion Prediction Analysis

The pharmacokinetics, drug-likeness, and medical chemistry features of 10 active compounds were predicted using SwissADME and were compared to reference clinical drugs for HNSCC patients including methotrexate, hydroxycarbamide, and erlotinib. Gastrointestinal absorption, blood–brain barrier permeability, uptake, and drug-likeness of most compounds were comparable to the current clinical drugs. The five liver drug enzymes in Table 4 are CYP1A2, CYP2C19, CYP2C9, CYP2D6, and CYP3A4. Whether a compound is a substrate of P-gp is the key to evaluating its efflux activity through the biofilms (Montanari and Ecker, 2015). The occurrence of typical multidrug resistance is closely related to drug efflux mediated by multidrug resistance proteins of the ABC transporter family (Locher, 2009). For example, P-gp (P-glycoprotein), ABCB1, and MDR1 (Chen et al., 1986). The drug-likeness index is whether the following requirements are met: Lipinski, Ghose, Veber, Egan, Muegge, and if two or more indexes are satisfied, the drug-likeness is good (Daina et al., 2017). Except for MOL000862, other active ingredients conformed to over two drug-likeness indicators. Among them, MOL008040 can penetrate the blood–brain barrier while MOL008039, MOL008041, and MOL000098 may cause the ache. MOL000285 is a P-gp substrate with low gastrointestinal absorption.

**TABLE 4:** The ADME analysis of active components.

ADME feature	Pharmacokinetics				Drug-likeness		Medical chemistry	
Mol ID	GI absorption	BBB permeant	P-Gp substrate	Inhibition of liver drug enzyme (5)	The number of druggability indicators	Bioavailability score	Pain	Synthetic accessibility
MOL000098	High	No	No	3	5	0.55	1	3.23
MOL005573	High	No	No	4	5	0.55	0	3.03
MOL000354	High	No	No	3	5	0.55	0	3.26
MOL008046	High	No	No	3	5	0.55	0	3.36
MOL008041	High	No	No	4	5	0.55	1	3.48
MOL008039	High	No	No	4	5	0.55	1	3.53
MOL008040	High	Yes	No	4	5	0.55	0	3.4
MOL000285	Low	No	Yes	2	2	0.85	0	6.4
MOL000279	High	No	No	0	3	0.55	0	6.53
MOL000862	High	No	No	0	1	0.17	0	6.55
MOL000796	High	No	No	0	2	0.55	0	6.61
Methotrexate	Low	No	No	0	2	0.11	0	3.58
Erlotinib	High	Yes	No	5	5	0.55	0	3.19
Hydroxycarbamide	High	No	No	0	3	0.55	0	2.06

## Discussion

HNC is the sixth most prevalent human cancer worldwide, which originates from the head and neck sites including the lips, pharynx, larynx, and tongue (Puram and Rocco, 2015). Rare specific diagnostic and prognostic-related markers for patients with HNC have been identified due to genetic heterogeneity and tumor diversity (Hammerman et al., 2015). Although there are current advancements in combined treatments for HNC patients, especially for metastatic and/or recurrent patients, the HNC patients’ outcomes have not changed much in recent years. Complications or side effects also aggravated the deterioration in patients’ life quality. Identification of safe and effective drugs to treat HNC is an urgent need. TCM that has been used as an alternative or complementary therapy in human malignancies showed high safety and efficacy.

In this study, we investigated the main active ingredients of YWLS and their potential mechanisms in treating HNSCC through network pharmacology and molecular docking studies. In the component-target-disease network, cerevisterol, (22e,24r)-ergosta-6-en-3beta,5alpha,6beta-triol, polyporusterone E, genkwanin, and polyporenic acid C were the top 5 components that have relatively high degrees, which were 228, 119, 113, 108, and 105, respectively. This suggests that they might be the main active ingredients for treating HNSCC. Cerevisterol and (22e,24r)-ergosta-6-en-3beta,5alpha,6beta-triol belong to steroids. Studies have found that steroids have anti-inflammatory, immunomodulatory (Calpe-Berdiel et al., 2007), and anti-cancer activities (Imanaka et al., 2008) in breast (Grattan, 2013), gastric (De Stefani et al., 2000), and lung (Mendilaharsu et al., 1998). An epidemiological study indicated that cancer risk reduction was positively correlated with plant sterol intake (Grattan, 2013). Cerevisterol has been reported to inhibit DNA polymerase alpha (Mizushina et al., 1999) and act as a potent inhibitor of NF-kappa B signaling activation (Kim et al., 2008). It was revealed that the transcription factor NF-κB is constitutively expressed in HNSCC tissues, which results in cancer cell proliferation, survival, invasion, metastasis, and poor survival of patients (Monisha et al., 2017). This indicated that cerevisterol might be a promising drug candidate to treat HNSCC, but further *in vitro* and *in vivo* experiments’ validation are necessary. Polyporusterone E which is isolated from PU belongs to cytotoxic steroids. Pharmacological studies showed that steroids exert anti-tumor effects mainly by preventing cancer cell proliferation and inducing cancer cell apoptosis (XiaoMei et al., 2017). Polyporusterone E has a dose-independent inhibitory effect in the cell proliferation of leukemia L-1210 (Ohsawa et al., 1992). As one of the major non-glycosylated flavonoids in many herbs, genkwanin exhibited a variety of pharmacological functions, such as anti-inflammatory, chemopreventive, and antibacterial activities. It exerted an anti-inflammatory effect by the regulation of the miR-101/MKP-1/MAPK signaling pathway and the downregulation of proinflammatory mediators such as TNF-a, IL-1B, and IL-6 (Gao et al., 2014). Polyporenic acid C is one of the lanostane-type triterpenoids, and it can induce cell apoptosis in human lung cancer cells through the death receptor-mediated apoptotic pathway and is a promising agent for lung cancer therapy (Ling et al., 2009). These data implied that the active ingredients might be the potential candidates against HNSCC.

We intersected the potential targets of active ingredients of YWLS and HNSCC-related genes. Four modules named AKT1, EGFR, TNF, and CYP3A4, respectively, were identified in the PPI network of the overlapped genes. Additionally, AKT1, EGFR, and TNF are the top 3 hub genes ranked by degrees. AKT1 is one of the serine-threonine protein kinase families and is a downstream target of phosphoinositide 3-kinase (PI3K). It was a key regulator in various cell processes including cell proliferation, survival, and angiogenesis in normal and tumor cells (Vivanco and Sawyers, 2002). Activated AKT was a frequent event in many cancers such as HNSCC (Marquard and Juecker, 2020). Constitutively phosphorylated AKT and elevated kinase activity were observed in a large fraction of HNSCC (Amornphimoltham et al., 2004), suggesting AKT signaling represented a clinically relevant target. Several AKT-targeting inhibitors have been developed. An AKT inhibitor, capivasertib (AZD5363), showed significant responses in patients with tumors that carried AKT1 E17K mutation (Kalinsky et al., 2021). Two distinct AKT inhibitors, ATP-competitive and allosteric inhibitors, are in clinical development, while the allosteric inhibitor MK-2206 has failed in single-agent activity in many clinical trials (Jsb and Uba, 2017). Another inhibitor, miransertib (ARQ 092), showed promising anti-tumor effects in early phase studies (Harb et al., 2015). We noted that AKT1 is a potential target for several active ingredients from AO, PU, and CR.

Increased EGFR expression, amplification, and low frequencies of single nucleotide variations/indels have been observed in HNSCC (Xu et al., 2017; Liu et al., 2020). The overexpression of EGFR is associated with decreased survival for patients (Rubin Grandis et al., 1998). The activation of EGFR acted as a stimulator of Ras-Raf-MAPK, PI3K/AKT/mTOR, and JAK-STAT signaling pathways that promote carcinogenesis through increased cell proliferation and survival (Zimmermann et al., 2006). Targeted therapy that is directed toward EGFR for HNSCC has attracted interest. Current anti-EGFR therapeutic strategies are to target the extracellular domain of the receptor with monoclonal antibodies such as cetuximab and panitumumab (Troiani et al., 2016) and the intracellular domain using tyrosine kinase inhibitors (TKIs) such as gefitinib, erlotinib, osimertinib, and afatinib (Fasano et al., 2014). However, the low rates of response or resistance are the main challenges (Chong and Jaenne, 2013). Recently, a crucial semisynthetic derivative of artemisinin named dihydroartemisinin (DHA) combined with osimertinib showed *in vitro* and *in vivo* cytotoxicity in HNSCC (Chaib et al., 2019). This might lead to a novel strategy of EGFR inhibitors combined with TCM due to less than 5% of HNSCC patients carrying EGFR mutations. EGF-stimulated recycling of EGFR can induce AKT phosphorylation through activating downstream signaling. EGFR and AKT1 have been revealed to play a synergistic tumor-promoting role to aggravate tumor progression in human lung cancer (Nishimura et al., 2015). In addition, TNF signaling has been shown to act as a tumor accomplice in HNSCC by decreasing tumor cell apoptosis or promoting an immune-suppressive tumor microenvironment (Sandra et al., 2002; Lu et al., 2011). For example, TNF-α was proved to promote invasion and metastasis by the NF-κB pathway in oral squamous cell carcinoma (Tang et al., 2017). TNF-α can also inhibit apoptosis by activation of AKT serine/threonine kinase in HNSCC (Sandra et al., 2002). It was noted that several ingredients of YWLS might target these proteins simultaneously to result in inhibitory effects in HNSCC, but further verification will make it convincing.

The KEGG pathway analysis indicated that these 212 targets were mainly enriched in PI3K-AKT, MAPK, RAS, EGFR tyrosine kinase inhibitor resistance, ErbB, PD-L1 expression, PD-1 checkpoint pathway in cancer, and TNF signaling pathways. Previous reports demonstrated that activation of these pathways is highly correlated with cell proliferation, survival, and metastasis in HNC carcinogenesis (Tang et al., 2017) (Marquard and Juecker, 2020) and drug resistance (Picon and Guddati, 2020). These pathways are potential therapeutic targets for HNSCC patients such as EGFR and PI3K/AKT signaling. Accordingly, pathway analysis indicated that the targets of each herb in YWLS were enriched in these signaling pathways. We found that increased expression of 42 genes was associated with decreased survival, which was consistent with previous evidence, such as EGFR, AKT1, SERPINE1, HSP90AA1, HSP90B1 (Fan et al., 2020), PLAU (Li et al., 2021), MAP2K1 (Jain et al., 2019), and CCND1 (Feng et al., 2011). Among these survival-related genes, 33 genes had higher expression in tumor tissues than in normal tissues like EGFR, AKT1, and HSP family genes, suggesting they could serve as diagnostic markers to distinguish normal from tumoral samples. An independent verification analysis has shown the consistent expression pattern of these targets in tumors versus normal tissues. In addition, ROC analysis showed that the AUC values of AURKA, PLK1, PLAU, MMP14, HSP90B1, SERPINE1, and CDK4 genes are greater than 0.9, exhibiting good performance. Several survival-related genes have been reported to be pharmacologic targets for solid tumors including HNSCC. CDK4/CDK6 inhibitors have been approved to treat breast and small cell lung cancer (Riess et al., 2021). CDK 4/6/7 inhibitors for HNSCC have been in preclinical and clinical applications. For example, palbociclib and ribociclib specifically inhibit CDK4 and CDK6, and abemaciclib selectively targets CDK4. CCND1 mutations and CDKN2A/B were the predictive biomarkers of response. Dual inhibition of EGFR and MAPK/CDK4/6 prevented oesophageal squamous cell carcinoma (OSCC) progression (Zhou et al., 2017). Therapeutic targeting of MAP2K1 in the MAPK pathway was a promising strategy for EGFR inhibitor (erlotinib)-resistant HNSCC patients (Jain et al., 2019). It was reported that Aurora kinases were potential targets to overcome EGFR inhibitor resistance in HNSCC, indicating that Aurora kinase A (AURKA) blockade might be a promising approach (Kim and Bandyopadhyay, 2021). PLK1 inhibitor could induce pyroptosis in OSCC to elevate cisplatin chemosensitivity (Wu et al., 2019). Inhibition of apoptosis signaling through BCL-xL and MCL-1 in HNSCC was a potential therapeutic option (Ow et al., 2020). These findings elucidated that the combination therapy with EGFR inhibitors might synergistically enhance the anti-HNSCC capacity and attenuate the resistance to EGFR therapy, and further experimental work is needed to verify this hypothesis.

The molecular docking study was used to validate the interactions between eight survival-related hub targets and their possible active components of YWLS. The compounds showed good binding scores with the corresponding targets such as AKT1, EGFR, PPARG, ESR1, and SRC. The ADME analysis was conducted to further assess the drug potentials of these compounds for HNSCC patients. Partial components exhibited comparable pharmacological characteristics with current clinical agents. These data indicated that these compounds might be used as potential therapeutic drugs to treat HNSCC.

## Conclusion

In summary, the potential therapeutic targets of active ingredients of YWLS for treating HNSCC were predicted by the network pharmacology studies, and molecular docking predicted the interactions between the active compounds and the related targets, and the drug-likeness properties of these compounds were further evaluated by the ADME analysis. The underlying mechanism of YWLS against HNSCC might be associated with PI3K-AKT, MAPK, and EGFR signaling pathways. These compounds might provide novel treatment strategies for HNSCC themselves or in combination with current molecular targeted therapies, and further verification by subsequent experiments is imperative.

## Data Availability

The original contributions presented in the study are included in the article/Supplementary Material; further inquiries can be directed to the corresponding authors. The data analyzed in this study are available in the following repositories: 1. TCGA: https://portal.gdc.cancer.gov/ 2. TCMSP: https://old.tcmsp-e.com/tcmsp.php 3. DisGeNET: https://www.disgenet.org/ 4. GeneCards: https://www.genecards.org/ 5. STRING: https://string-db.org/ 6. RCSB PDB: https://www.rcsb.org 7. PubChem: https://pubchem.ncbi.nlm.nih.gov/ 8. SwissTargetPrediction: http://www.swisstargetprediction.ch/ 9. SwissADME: http://www.swissadme.ch/.

## Abbreviations

HNC, head and neck cancer; HNSCC, head and neck squamous cell carcinoma; YWLS, Yinchen Wuling San; PU, Polyporus umbellatus; AO, Alisma orientale; PC, Poria cocos; AMK, Atractylodes macrocephala koidz; CR, Cinnamomi ramulus; ASH, Artemisiae scopariae herba; DEGs, differentially expressed genes; TCGA, The Cancer Genome Atlas; FPKM, Fragments Per Kilobase of transcript per Million mapped reads; TCMSP, traditional Chinese medicine systems pharmacology database and analysis platform; PPI, protein–protein interaction; MCODE. molecular complex detection; BP, biological process; CC, cellular component; MF, molecular function; KEGG, Kyoto Encyclopedia of Genes and Genomes; OS, overall survival; ADME, absorption, distribution, metabolism, and excretion. OSCC, oral squamous cell carcinoma; PCA, principal component analysis; ROC, receiver operating characteristic; and AUC, area under the curve
