# Supplementary figures and images for: Identification of Molecular Targets and Potential Mechanisms of Yinchen Wuling San Against Head and Neck Squamous Cell Carcinoma by Network Pharmacology and Molecular Docking
Source: Front Genet. 2022 Jul 6;13:914646. doi: 10.3389/fgene.2022.914646 (PMC9306494; doi:10.3389/fgene.2022.914646)

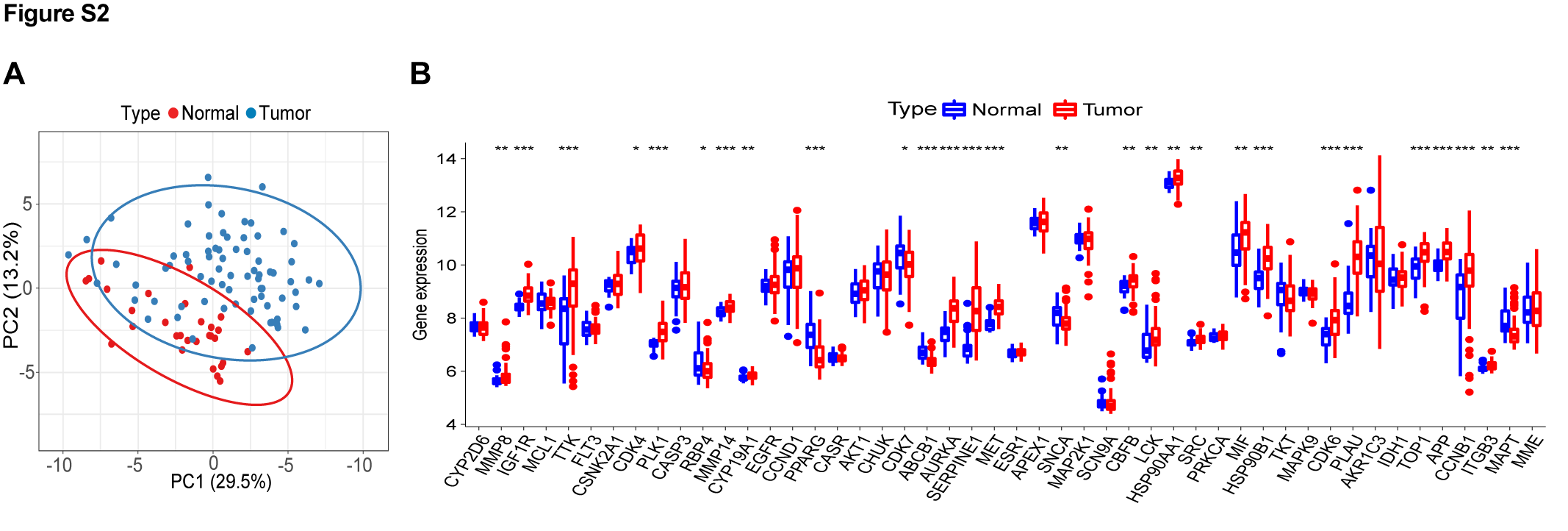

Supplement: Supplementary file 3 [file Image2.TIF]

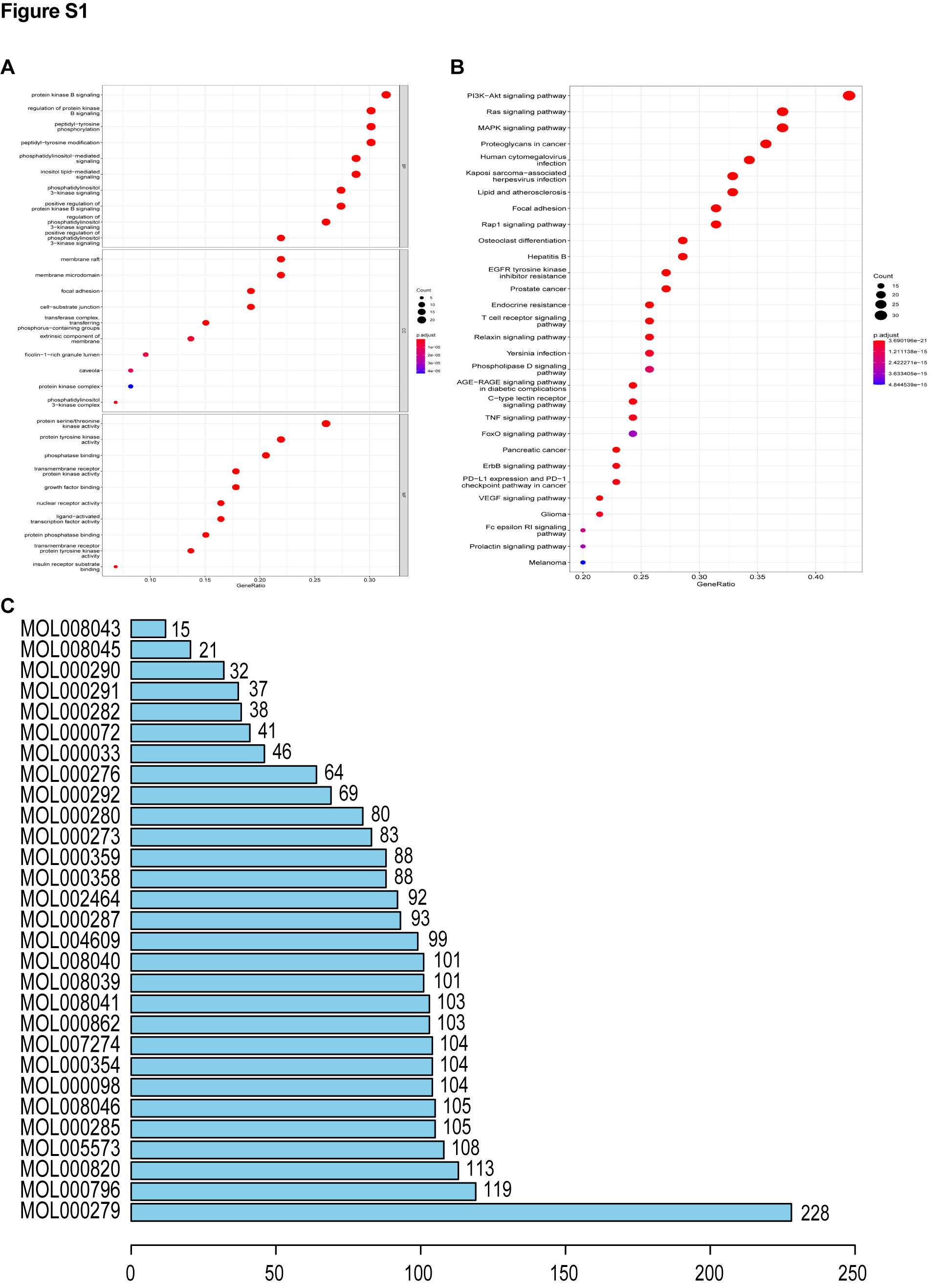

Supplement: Supplementary file 4 [file Image1.TIF]
